# Home-Based Spirometry Telemonitoring After Allogeneic Hematopoietic Cell Transplantation: Mixed Methods Evaluation of Acceptability and Usability

**DOI:** 10.2196/29393

**Published:** 2022-02-07

**Authors:** Ajay Sheshadri, Sukh Makhnoon, Amin M Alousi, Lara Bashoura, Rene Andrade, Christopher J Miller, Karen R Stolar, Muhammad Hasan Arain, Laila Noor, Amulya Balagani, Akash Jain, David Blanco, Abel Ortiz, Michael S Taylor, Alex Stenzler, Rohtesh Mehta, Uday R Popat, Chitra Hosing, David E Ost, Richard E Champlin, Burton F Dickey, Susan K Peterson

**Affiliations:** 1 Department of Pulmonary Medicine The University of Texas MD Anderson Cancer Center Houston, TX United States; 2 Department of Behavioral Sciences The University of Texas MD Anderson Cancer Center Houston, TX United States; 3 Department of Stem Cell Transplantation The University of Texas MD Anderson Cancer Center Houston, TX United States; 4 Monitored Therapeutics, Inc Dublin, OH United States

**Keywords:** allogeneic hematopoietic cell transplantation, home-based spirometry, acceptability, usability, mixed methods evaluation, patient perspectives, spirometry, feasibility, mHealth, home-based, remote care, respirology, pulmonary medicine, mobile phone

## Abstract

**Background:**

Home-based spirometry (HS) allows for the early detection of lung complications in recipients of an allogeneic hematopoietic cell transplant (AHCT). Although the usability and acceptability of HS are critical for adherence, patient-reported outcomes of HS use remain poorly understood in this setting.

**Objective:**

The aim of this study is to design a longitudinal, mixed methods study to understand the usability and acceptability of HS among recipients of AHCT.

**Methods:**

Study participants performed HS using a Bluetooth-capable spirometer that transmitted spirometry data to the study team in real time. In addition, participants completed usability questionnaires and in-depth interviews and reported their experiences with HS. Analysis of interview data was guided by the constructs of performance expectancy, effort expectancy, and social influence from the Unified Theory of Acceptance and Use of Technology model.

**Results:**

Recipients of AHCT found HS to be highly acceptable despite modest technological barriers. On average, participants believed that the HS was helpful in managing symptoms related to AHCT (scores ranging from 2.22 to 2.68 on a scale of 0-4) and for early detection of health-related problems (score range: 2.88-3.12). Participants viewed HS favorably and were generally supportive of continued use. No significant barriers to implementation were identified from the patient’s perspective. Age and gender were not associated with the patient perception of HS.

**Conclusions:**

Study participants found HS acceptable and easy to use. Some modifiable technical barriers to performing HS were identified; however, wider implementation of pulmonary screening is feasible from the patient’s perspective.

## Introduction

### Background

Despite improvements in the recognition and treatment of bronchiolitis obliterans syndrome (BOS) after allogeneic hematopoietic cell transplantation (AHCT), outcomes remain poor [1]. The risk of death after BOS correlates with the severity of airflow impairment at diagnosis [1,2], and recipients of AHCT with severe airflow obstruction at BOS diagnosis have increased nonrelapse mortality compared with recipients of AHCT without BOS [3]. Early BOS presents as lymphocytic bronchiolitis and may be more amenable to treatment [4], whereas late BOS presents as airway fibrosis and is generally treatment refractory [5]. As BOS is a disease with the potential for rapid progression [6], prompt diagnosis may improve therapeutic outcomes.

Home-based spirometry (HS) has been an effective strategy for diagnosing early BOS in recipients of lung allograft and is standard practice for posttransplantation monitoring of lung function. Several studies have documented excellent adherence and proficiency with forced spirometric maneuvers [7-10]. Although fewer studies have been conducted in recipients of AHCT [11-13], the progression of BOS related to graft-versus-host disease (GVHD) may be slowed or halted in recipients of AHCT in whom airflow obstruction is promptly recognized and treated [1]. As a result, despite the lower incidence of BOS in recipients of AHCT than chronic lung allograft rejection [14-16], which occurs in most recipients of a lung transplant, given sufficient time [17], a spirometric telemonitoring program may be valuable for diagnosing BOS in recipients of AHCT at an earlier stage and preventing progression.

### Objective

Recipients of AHCT may face barriers to performing HS. First, psychosocial burnout and fatigue are common symptoms among recipients of AHCT and may be more prevalent early in the course after transplantation [18,19]. Therefore, recipients of AHCT may not be willing to perform routine HS measurements during this time, when screening for BOS could be of significant benefit [20]. Second, until recently, real-time data collection was not possible, and HS data were often evaluated by less efficient methods such as by mail or landline phone connections [11,12]. By leveraging the widespread use of smartphones and wireless connectivity [21], we seek to implement an HS pilot program through which spirometry data could be delivered in near real time by wireless networks, allowing for scalable, efficient telemonitoring. We previously reported the technical feasibility of performing home spirometry in recipients of AHCT [22]. Here, we seek to assess the usability and acceptability of HS among recipients of AHCT using mixed methods–patient-reported outcome (PRO) surveys and in-depth interviews.

## Methods

### Study Overview

**Figure 1:**
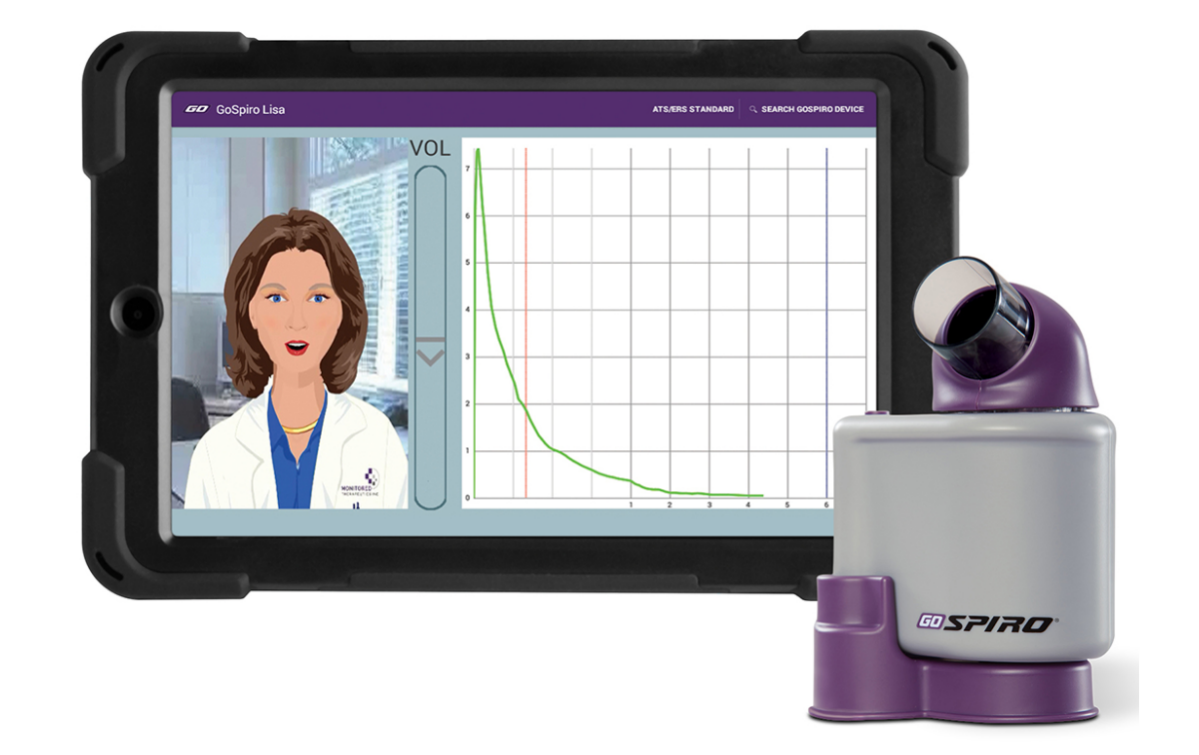
GoSpiro Bluetooth-compatible home spirometer (forefront) and the GoHome wireless tablet (background).

This paper describes the results of a pilot feasibility study of HS. The full details of the study protocol have been previously reported [22]. Briefly, at enrollment, participants were given a Bluetooth-compatible home spirometer (GoSpiro, Monitored Therapeutics Inc), which communicates wirelessly with patients’ smartphones through an app or custom tablets (GoHome, Monitored Therapeutics Inc; Figure 1). Participants with tablets could additionally take advantage of an automated coaching algorithm which gave instructions to encourage maximal expiratory flow and sufficient expiratory time. Spirometric and questionnaire data were subsequently transmitted to a remote electronic server via an internet-based portal. Participants were instructed to perform 3 maneuvers per session up to 3 times per week. Those who were unable to remain adherent to the study protocol for a sufficient period to generate a baseline forced expiratory volume in 1 second (FEV_1_) measurement (ie, they did not perform at least one technically acceptable HS maneuver on 6 separate days within the first 2 weeks) were removed from the study. Participants were instructed to continue measuring until at least 1 year after the transplant or approximately 9 months after enrollment. One week of nonadherence to the study protocol resulted in weekly phone calls or emails to the patient as a reminder to resume measurements at their convenience.

### Participants

We consented and enrolled English-speaking adult recipients of AHCT who were seen at an AHCT survivorship clinic at approximately 100 days after transplantation between October 2016 and June 2018. Patients were enrolled in a longitudinal cohort study lasting up to 9 months, involving a baseline visit followed by remote measurement of key clinical and PRO variables. Demographic and clinical data were also collected from electronic health records and institutional databases. To gather information on user experience, participants completed web-based surveys on usability and acceptability at 1-, 3-, 6-, and 9-month follow-ups using REDCap (Research Electronic Data Capture) [23] and completed one-on-one telephone interviews at 1-, 3-, and 6-month follow-ups. The study was approved by the MD Anderson institutional review board (2015-0990).

### Quantitative Data Collection

At 1, 3, 6, and 9 months, participants were asked to answer follow-up questions about the HS device, app design, its features, its overall usefulness, satisfaction, their intention to recommend spirometry to other recipients of AHCT, the acceptability of the tablet and mobile app, its impact on health management, and suggestions to improve the spirometer in the future. Usability testing was conducted using a structured questionnaire to collect responses to 14 Likert-style questions scored on a 5-point scale ranging from 0 (not at all) to 4 (extremely), as well as 5 open-ended questions. Example questions include “Overall, how satisfied were you with the GoSpiro Home Spirometer?” and “How useful do you believe the GoHome tablet and GoSpiro app is in helping you to manage your symptoms related to your stem cell transplantation?”

Patient engagement with remote monitoring was measured using the patient activation measure (PAM), which comprises 13 items with strong psychometric properties [24]. Items are focused on constructs of confidence, beliefs, knowledge, and skills about managing one’s health, which respondents can answer with degrees of agreement or disagreement (eg, “I know how to prevent problems with my health” and “I am confident that I can tell a doctor my concerns, even when he or she does not ask”). The measure is scored on a theoretical 0 to 100 scale, with higher scores reflecting a developmental progression from passive receipt of care toward greater activation.

### Qualitative Data Collection

We conducted one-on-one telephone interviews with participants at 1, 3, and 6 months of the study period. Interviews followed a semistructured interview guide designed to elicit participants’ views on the usability (functionality, navigation, and interactivity) and acceptability of HS. Open-ended qualitative questions included the following:

- “How has using the HS Spirometer affected your health in general?”
- “Is there anything you do not like about the HS Spirometer or that made you not want to use that device?”
- “How helpful do you think it would be for other patients like you to use the HS spirometer?”

Interviews were audio recorded, professionally transcribed, and redacted before directed qualitative content analyses.

### Theoretical Model

The Unified Theory of Acceptance and Use of Technology (UTAUT) model was originally developed as a conceptual framework to explain individuals’ intention to adopt and use technological innovations [25]. In this study, we drew from this theory to describe the applicability and likelihood of using the HS device among recipients of AHCT. UTAUT is a widely used model of information technology adoption and has been used to examine the adoption of various technologies in different contexts [26]. The following key theoretical constructs from the UTAUT model guided the analysis of in-depth interview data: (1) performance expectancy, (2) effort expectancy, and (3) facilitating conditions. The construct of social influence from the original model was eliminated as it was not applicable to this study. We also sought to identify potential contextual factors that might affect home spirometer use based on moderating characteristics identified by UTAUT (age, gender, experience, and voluntariness).

### Data Analysis

#### Quantitative Data Analysis

We summarized demographic, clinical, and PROs as mean (SD) or number (percentage). Differences between groups were compared using chi-square analysis for categorical variables or 2-tailed *t* tests for continuous variables. Participant ratings for each usability question were reported as the mean score per item (scale 0-4).

#### Qualitative Data Analysis

Initial content analysis via line-by-line coding, a form of open coding, was performed by 2 coders independently to identify emergent codes. The coders then met to debrief and discuss emergent codes derived from the process. Codes were discussed and clarified between the 2 analyses, and adjustments were made until intercoder consensus was achieved, which is an approach that ensures rigor. Discrepancies were resolved by discussion, and quotations illustrating the main themes were identified during the coding process. Secondary content analyses were then performed by applying the UTAUT as a semistructured framework. Atlas.ti (version 8.0; ATLAS.ti Scientific Software Development GmbH) qualitative data analysis software was used for the coding and content analysis.

#### Mixed Methods Integration

The value of mixed methods research lies in the meaningful integration of qualitative and quantitative components. We used merging integration, which comprised comparing qualitative findings with respect to PAM scores and survey responses with usability questions and determining whether the qualitative findings did or did not support, or expand, our understanding of the scores [27].

## Results

### Study Sample

**Figure 2:**
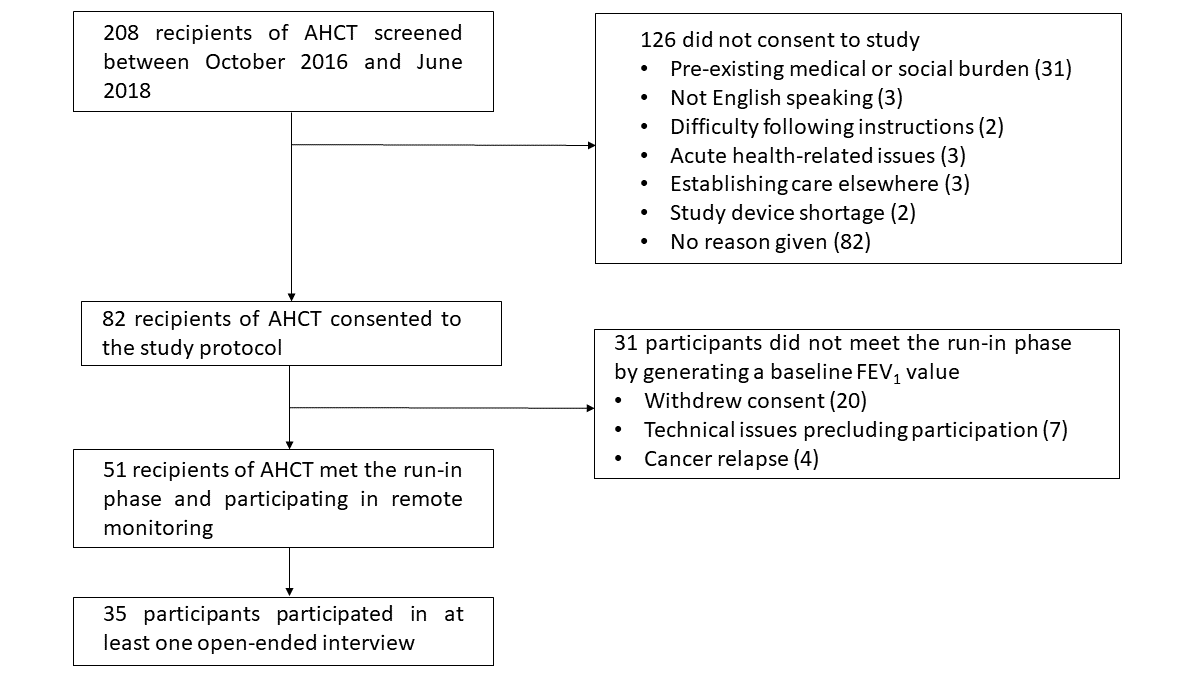
Study enrollment flowchart. AHCT: allogeneic hematopoietic cell transplant; FEV_1_: forced expiratory volume in 1 second.

We screened 207 patients for study participation (Figure 2). Of the 207 patients, 82 (39.6%) completed the baseline assessment and received training to use the spirometer. Of these 82 patients, 51 (62%) performed spirometry and registered baseline values for FEV_1_ and were considered to be adequately familiar with HS to complete qualitative and quantitative measures on HS use. Table 1 provides the demographic characteristics of all study participants, whereas Table 2 provides the demographic characteristics of participants who completed the survey and interview at each follow-up time point. Participants who did not remain in the study long enough to register baseline values for FEV_1_ (ie, did not perform at least six spirometric maneuvers on separate days within the first month) did not differ significantly from participants included in the study with respect to any demographic characteristics (age, race, ethnicity, and sex). Of the 51 patients, usability surveys were completed by 27 (53%), 22 (43%), 24 (47%), and 15 (29%) participants at the 1-, 3-, 6-, and 9-month follow-ups, respectively. Of the 51 patients approached for recruitment over the course of the study, 35 (69%) recipients of AHCT completed one or more telephone interviews: 3 (9%) completed all 3 interviews, 9 (26%) completed 2 interviews, and 23 (66%) completed 1 interview, with an overall response rate of 69%. Participants who completed open-ended interviews were predominantly older, with a median age of 58 (range 23-75) years, non-Hispanic White, and male. PAM scores (ranging between 80.9 and 86.8) indicated a relatively high level of engagement in the management of self-care while living with a chronic illness (Table 2).

**Table 1:** Characteristics of the overall study cohort (N=51).

Variable			Values
Age (years), median (IQR)			55 (41-64)
**Sex, n (%)**			
	Male	17 (33)	
	Female	34 (67)	
**Race, n (%)**			
	White	47 (92)	
	Person of color	4 (8)	
**Underlying malignancy, n (%)**			
	Acute myeloid leukemia or myelodysplastic syndrome	25 (49)	
	Acute lymphoblastic leukemia	4 (8)	
	Chronic lymphocytic leukemia	4 (8)	
	Chronic myeloid leukemia	7 (14)	
	Lymphoma	6 (12)	
	Myeloma or plasma cell disorder	3 (6)	
	Myelofibrosis	2 (4)	
**Acute GVHD^a^ before enrollment, n (%)**			
	Yes	39 (76)	
	No	12 (24)	
**Chronic GVHD at enrollment, n (%)**			
	Yes	18 (35)	
	No	33 (65)	
Baseline PAM^b^ score (IQR)			66 (56-80)
Baseline FEV_1_^c^, % predicted (IQR)			96 (83-106)
Baseline FVC^d^, % predicted (IQR)			91 (80-98)
Baseline FEV_1_/FVC ratio (IQR)			79 (76-84)

^a^GVHD: graft-versus-host disease.

^b^PAM: patient activation measure.

^c^FEV_1_: forced expiratory volume in 1 second.

^d^FVC: forced vital capacity.

**Table 2:** Characteristics of participants who provided open-ended interviews (N=51).

Variable				1 month (n=25)		3 months (n=22)		6 months (n=24)		9 months (n=15)
**Quantitative data (usability survey)**										
	Age (years), median (range)			59 (32-74)		60 (33-72)		62.5 (40-74)		64 (33-72)
	**Sex, n (%)**									
		Male	14 (56)		12 (55)		15 (63)		10 (67)	
		Female	11 (44)		10 (46)		9 (38)		5 (33)	
	**Race, n (%)**									
		Asian	0 (0)		1 (5)		0 (0)		0 (0)	
		Black	1 (4)		0 (0)		0 (0)		0 (0)	
		White	24 (96)		20 (91)		24 (100)		15 (100)	
		Other	0 (0)		1 (5)		0 (0)		0 (0)	
	**Ethnicity, n (%)**									
		Hispanic	1 (4)		2 (9)		3 (13)		1 (7)	
		Non-Hispanic	23 (92)		19 (86)		21 (88)		14 (93)	
		Unknown	1 (4)		1 (5)		0 (0)		0 (0)	
	Patient activation measure, mean (range)			86.4 (67.3-100)		84.3 (63.5-100)		86.8 (73.1-100)		80.9 (57.7-100)
**Qualitative data (semistructured interview)**										
	Completion rate			15 (29.4)		14 (27.5)		21 (41.2)		N/A^a^

^a^N/A: not applicable.

### Usability Ratings

Overall, participants rated HS as highly usable, and usability remained high throughout the study period (Table 3). On average, participants believed that HS was helpful in managing symptoms related to AHCT (scores ranging from 2.22 to 2.68 on a scale of 0-4) and for early detection of health-related problems (score range 2.88-3.12). They were also willing to recommend the spirometer to other recipients of AHCT (score range 2.65-3.16) and were satisfied with HS overall (score range 2.31-3.53). The only negatively worded item about their illness interfering with spirometer use received low scores (range 0.62-0.88), indicating that AHCT was not a barrier to using the spirometer. Similarly, patients also rated the GoHome tablet and GoSpiro app as highly usable, satisfactory, and helpful.

**Table 3:** Usability questionnaire scores (N=51).

Usability items			1 month (n=25)					3 months (n=22)					6 months (n=24)					9 months (n=15)		
			Value, mean (SD)^a^		Positive response, n (%)		Value, mean (SD)			Positive response, n (%)		Value, mean (SD)			Positive response, n (%)		Value, mean (SD)			Positive response, n (%)
**GoSpiro home spirometer**																				
	Illness interfered with ability to use the home spirometer	0.88 (1.4)		3 (12)		0.82 (1.05)			1 (5)		0.62 (0.97)			2 (8)		0.79 (1.18)			1 (7)	
	Ease of use	2.19 (1.52)		12 (48)		2.55 (1.14)			14 (64)		2.67 (1.34)			16 (67)		2.43 (1.28)			9 (60)	
	Helpful in managing symptoms related to stem cell transplantation	2.22 (1.42)		11 (44)		2.68 (1.17)			14 (64)		2.54 (1.32)			13 (54)		2.33 (1.29)			6 (40)	
	Helpful for early detection of health-related problems	2.88 (1.22)		16 (64)		3 (0.93)			17 (77)		3.12 (0.95)			20 (83)		3 (1.24)			11 (73)	
	Belief that GoSpiro home spirometer helps health care providers monitor illness	2.95 (1.31)		17 (68)		3.04 (1.13)			18 (82)		2.92 (0.97)			18 (75)		3.14 (1.17)			11 (73)	
	Using the GoSpiro home spirometer gives a feeling of security	2.44 (1.42)		14 (56)		2.77 (0.97)			16 (73)		2.75 (1.15)			16 (67)		2.8 (0.94)			9 (60)	
	Willingness to continue using for up to 2 years	2.35 (1.23)		11 (44)		2.18 (1.3)			10 (45)		2.25 (1.48)			13 (54)		2.26 (1.71)			8 (53)	
	Willingness to recommend to other patients who receive a stem cell transplantation	2.65 (1.25)		13 (52)		2.9 (1.02)			14 (64)		3.16 (0.96)			19 (79)		3 (1.13)			10 (67)	
	Overall satisfaction	2.4 (1.39)		13 (52)		2.32 (1.09)			12 (55)		2.79 (1.1)			15 (63)		3.53 (1.3)			9 (60)	
**GoHome tablet and Go Spiro app**																				
	Ease of use	2.22 (1.55)		13 (52)		2.59 (1.05)			15 (68)		2.92 (1.05)			16 (67)		2.53 (1.25)			9 (60)	
	Helpful in managing symptoms related to stem cell transplantation	2.34 (1.47)		12 (48)		2.27 (1.16)			11 (50)		2.75 (1.18)			15 (63)		2.33 (1.34)			8 (53)	
	Willingness to recommend to other patients who receive a stem cell transplantation	2.65 (1.28)		14 (56)		2.72 (0.94)			15 (68)		3.16 (0.96)			19 (79)		2.86 (1.4)			9 (60)	
	Overall satisfaction	2.26 (1.53)		11 (44)		2.38 (0.97)			13 (59)		2.66 (1.05)			12 (50)		2.4 (1.18)			7 (47)	
**Automated** **coaching**																				
	Overall satisfaction	2.59 (1.43)		14 (56)		2.45 (1.36)			12 (55)		2.87 (1.14)			15 (62)		2.93 (1.32)			11 (73)	

^a^Scores range from 0=not at all to 4=extremely; scores of 3 or 4 were considered positive responses.

### Qualitative Findings

Participant responses around usability and acceptability of HS as well as contextual factors that modify these responses are summarized in Table 4 and discussed below. Qualitative results are organized by the constructs of the UTAUT model, which represent overarching messages emerging out of the cross-sectional and narrative analysis of the interviews over the three time points: 1, 3, and 6 months after transplantation.

**Table 4:** Example quotes from study participants mapped within constructs of the Unified Theory of Acceptance and Use of Technology construct model.

Unified Theory of Acceptance and Use of Technology construct			Example quotes
**Performance expectancy**			
	Perceived usefulness	“I think it’s a valuable tool and I see benefits for me and I’m sure others would. I think it’s easy to operate and I’m pleased with it. I think it’s doing me some good.” “I think it would be very helpful, just because they’re so many unexpected complications with the stem cell transplant and I think that...it would be very easy to use to get that information.” “I think it’s a great product and it’ll help a lot of other people. It’s also going to help me if there’s anything developing that you need to know about in advance.” “Well, I think it’s a good system. I think it should be considered as part of the standard treatment for transplant patients. I’m glad to have it and it doesn’t take a lot of time, it doesn’t take a lot of energy and so it’s something that’s reassuring, and I don’t mind doing it at all”	
	Outcome expectations	“If I develop pulmonary GVHD, we’re likely to catch it much sooner. It can be interrupted at an earlier stage well before it damages my lungs too much.” “I think it’s helped me to try to do better to maintain...better health because I will take my measurements and I always try to like keep higher measurements than before or try to maintain it at the same level.” “From the beginning, I kind of thought that, ‘well, it was a good reading,’ but yeah what does that mean? That was a little higher—does that mean that’s good or bad? I mean, it’d be nice to know that you’re still on the right track.” “No. I mean basically, I do the same things that I was doing before, and I haven’t changed anything”	
	Relative advantage	“Well, I can tell if something’s wrong with my lungs easily, but if there’s something that I’m not seeing, then maybe [it] makes me feel good to know that they may be able to see a change in pressure...that I can’t tell. Identify graft versus host disease in my lungs before I know it” “It’s very easy and you can do it at home, it’s a lot easier than going to someplace and get it done professionally...this is reassuring to me that I’m [not] going downhill with GVHD.”	
**Effort expectancy**			
	Ease of use	“Yeah, I’ve got it down where I can do it quickly to the cell phone and the GoSpiro device, they went up quickly on Bluetooth and light turns green two, three times. And using the technique that they showed me and I would say it’s very quick, very easy.” “I mean it’s pretty quick. I usually do it around dinner time, I’ll pull it up, it takes, maybe a minute totally set it up. I take a minute and a half and I’m done with everything.” “Yeah. I’ve kind of made it into a game to see if I can get up to eight each time and I’m usually right about eight, upper sevens or low eights each time I do it. But yeah, it’s almost like making them into a little game, competition, see I can do better than I did the day before. Now there’s certainly a technique blowing into it and it took me a while to learn the technique” “It’s part of the routine getting dressed, do breathing exercise three times a week, and it’s not a problem whatsoever, it’s been easy.” “I think the phone is pretty convenient. I don’t really see an issue with that.”	
	Complexity	“So [charging] is very quirky I find, but, it’s not difficult to work with. It’s just difficult to get the three tests all in a row...it takes a little time, and it’s not something I can do in 10 minutes or so because I have to play with it to get it to work all the time.” “Well, I wanted to use it and it wouldn’t work. So we figured it wasn’t lying on the charger correctly, it was bent somehow so it would not lie on the charger correctly” “I continue thinking that the charging port needs to be a little bit longer. The cord is really short, not only that but having, maybe possibly indicator like for charging on the spirometer itself—red while it charged and then it turns green when it’s fully charged” “But I was doing it and apparently it was working, the little Avatar said your measurement has been recorded. So I thought everything was right and then I received a call saying if I wanted to continue with this study. I was like why would you say that and they said because you only have done one measurement during the whole week in a month.” “The communication between the Spiro and the tablet was broken very frequently. I have to repeat several times the process of the measurement, and then I was not able to do it, nothing. The technical things are the problem.”	
**Facilitating conditions**			
	Portability	“[I] take it apart and just set it in a suitcase. The battery lasts for the duration of my travel, so I just take the device itself, so it doesn’t take up a lot of room and it travels well.”	
	Reminders and positive reinforcement	“The bottom line is that you need to have some similar goal system set up, so the participants continue to be interested in doing it. I think it’s just natural human nature that if you’re being rewarded for whatever you’re doing as simple as, doing a great job or hey you’re doing great, your scores are continuing to being in pain or whatever, some kind of feedback to encourage you to do at the next time, I think would be helpful.”	

### Performance Expectancy

Perceived expectancy and plans to use the HS device were operationalized through three subconstructs of the UTAUT model: *perceived usefulness*, *outcome expectations*, and *relative advantage*.

The perceived usefulness of HS was ascertained from the patients’ summative experiences and perceived benefits gained from using the instrument. In agreement with survey responses, most participants felt that HS would be helpful in detecting early complications with stem cell transplantation, including GVHD, and that it should be considered part of the standard treatment for recipients of AHCT. The spirometer was believed to be highly useful and easy to use.

*Outcome expectations* are expectations regarding the impact of using HS on health and health behavior. Most participants expected that regular HS use would enable them to better maintain their health by retaining consistent pulmonary function. They reported self-competition and goal setting as factors that motivated them to continue HS use. In addition, the knowledge that a member of the study team was regularly monitoring their lung function and would intervene if needed was reassuring to the study participants. One of the expectations *not met* by the HS device was its inability to provide real-time feedback and interpretation of pulmonary function measurements. None of the participants reported making any health behavior change as a result of using HS. These data are supported by the survey results, where participants expressed a strong belief that HS allowed providers to monitor the illness.

*Relative advantage* relates to the degree to which an innovation is perceived as being better than using its precursor. In this study, participants believed that the benefits of using HS vastly outweighed the use of clinic-based spirometry, and this belief was a prominent positive influence for their continued completion of spirometric measurements. They were aware of the possible complications of AHCT, including GVHD, and thus, the ability to monitor pulmonary health without having to go to the hospital was deemed immensely advantageous. These interview results were supported by survey findings where use was reported to provide a feeling of security and help manage symptoms related to transplantation.

### Effort Expectancy

Effort expectancy is defined as the degree of ease associated with the use of a system with two subconstructs: *ease of use* and *complexity.* Many participants reported that the HS device was easy to use and found the overall HS interface to be intuitive, approachable, and easy to use and synchronize to their smartphone or tablet. Many reported that they could complete the measurements within a few minutes, had turned the measurements into a routine (eg, completing measurements before dinner or after waking up), or had gamified the process to keep themselves motivated. Self-competition was seen as a prominent positive influence on adherence to use. However, others experienced technical difficulties when using the device and continued to experience technical difficulties when using the HS device over the study period. They reported issues with charging the battery, connectivity between devices, and issues with uploading data from the HS device to the server. The consequent failed attempts at recording measurements were frustrating for some participants.

### Facilitating Conditions

Most reported that the HS device was easy to maintain and did not report problems with cleaning the mouthpiece and storing the HS device. We specifically asked about the portability of the HS device during interviews, and portability was found to be a facilitating condition for continued use. In fact, participants found the HS device to be portable enough that they reported taking it with them on vacations. On some occasions, participants who were hospitalized or very tired from treatment were not able to complete the HS measurements. This qualitative observation is also reflected in the survey, where participants rarely felt that their illness interfered with HS use.

Participants had suggestions for improvements to facilitate greater use of the device in the future. They suggested adding a trends feature in the app to monitor pulmonary health over time, reminders for weekly use, and positive reinforcements to encourage continued use. Some also suggested that a more comfortable nose clip would be helpful, and reminders to put on the clip before measuring lung function would also be appreciated.

Overall, thematic weaving of results from participants’ survey responses and their answers to open-ended interview questions showed a good fit between qualitative and quantitative data and deepened our understanding of the findings. Qualitative findings confirmed the survey responses and provided further insight into the survey responses through specific examples or explanations offered in the interviews. Mixed methods integration showed no apparent relationship between participants’ responses and their age or sex. It was not possible to infer a relationship between participants’ engagement in their health care and their use and adherence to HS because of the limited variance in PAM scores in our sample.

## Discussion

### Principal Findings

The aim of this pilot feasibility study was to investigate the acceptability and usability of an HS program among patients who had undergone AHCT using mixed methods. Overall, we found that recipients of AHCT generally found routine, unsupervised, patient-performed HS to be acceptable, despite some technological barriers. Spirometer use was viewed favorably by participants, with enthusiasm for continued use. Although some barriers to performing HS were identified, most were amenable to improvement, and patients were forthcoming with solutions on how to alter their personal use of HS to overcome barriers. Some barriers were unique to this study and the specific devices used, such as periodic difficulty in transmitting data from the HS device to the server. These types of barriers may commonly occur when new technology is introduced in a patient care setting. The results of this study can inform the adoption and implementation of HS on a wider scale and potentially address the need for innovative remote patient monitoring technologies highlighted by the COVID-19 pandemic.

HS is a promising emerging technology that allows for longitudinal monitoring of lung function in high-risk patients. Ideal conditions for HS include screening for diseases that have variable times of onset or monitoring those with variable patterns of progression, particularly when clinic-based monitoring is not practical because of the necessary frequency of testing. This strategy has been successful in recipients of a lung transplant for the monitoring of chronic lung allograft dysfunction, usually in the form of BOS [7-10]. More recently, this strategy has been adopted for patients with idiopathic pulmonary fibrosis, a disease with variable tempos and patterns of progression [28]. Patients with pulmonary fibrosis value home monitoring of symptoms and lung function [29], and these remote monitoring modalities are particularly relevant during the current COVID-19 pandemic, where in-person testing may be infeasible or unsafe [30].

Prompt diagnosis and treatment of BOS are imperative to reduce mortality or permanent loss of lung function [1]. HS is an attractive option for monitoring recipients of AHCT at risk for BOS as BOS often occurs later in the course of a transplant when direct patient contact is less frequent [20,31]; symptoms may often be insidious, even in the setting of significant pulmonary impairment [32]; and the time to onset of BOS and trajectory of impairment after BOS onset is variable [1]. By using an HS program that leveraged cloud-based data delivery to allow participants to perform spirometry anywhere with a cellular connection, we removed major barriers to performing spirometry and interpreting results in a timely fashion [33]. Furthermore, pulmonary impairments could be recognized quickly, allowing for prompt coordination of clinical care. However, none of the patients in our study developed BOS. This may be as we studied recipients of AHCT who were approximately 100 days after hematopoietic cell transplant up to 1 year after hematopoietic cell transplant; BOS more typically occurs in the second year after AHCT, although earlier cases have a poorer prognosis [20]. In addition, we did not take advantage of enriching our cohort with high-risk cases, such as those with high-risk chronic GVHD or prior lung complications [34]. Turner et al [34] found that in an enriched cohort, approximately 25% of patients developed BOS. Therefore, HS initiation would likely be more effective if started later in the course of AHCT in high-risk recipients.

For the most part, participants confirmed that routine HS was feasible and straightforward to perform. This observation was validated by the fact that, on average, participants completed measurements at 69% of possible weeks during the study period, and 94% of weeks with measurements had at least one technically sound loop despite the lack of a face-to-face encounter with a respiratory therapist [22]. As highlighted in our qualitative interviews, participants were highly motivated to participate in our home spirometry program. However, as we noted in our prior work, participants often had urgent health issues that interfered with their participation in home spirometry, as we previously reported [22]. In addition, although we considered missed measurements as nonadherence, it is important to note that some of the observed nonadherence was also because of technical errors with data transmission. The mixed methods design, which combined longitudinal interviews with periodic follow-up contact by study staff, enabled us to identify these technical errors in a timely manner.

Many participants valued knowing their HS measurements as they provided insight into the rapidity of their disease progression. Implementation of immediate feedback could serve those participants who understood how to interpret spirometry data by providing positive feedback for engagement while easing frustrations for those who did not understand how to interpret spirometry data by explaining what these measurements mean in relation to their prior data. Participants who reported not being able to interpret their spirometry data suggested that more immediate feedback on daily measurements would be helpful in understanding progression. Our study did not implement the interpretation of pulmonary function tests in real time for patients, which could improve patients’ understanding of their lung function. Interestingly, despite the lack of immediate feedback, our prior work suggested that technical proficiency was maintained or even slightly improved over the duration of the study [22]. Participants used these to set personal FEV_1_ goals and to self-monitor. They also felt that extrinsic motivators would improve adherence, for example, feedback from the study team that their lungs were doing well. Including extrinsic patient motivators in the design of HS, and other remote biometric monitoring platforms, may be an important feature for increasing engagement and adherence. Certain factors were found to hinder spirometer use, such as technical issues with charging and synchronization with cloud data. These barriers to use did not dissipate over time. Efforts to address these technical issues should be a priority for investigators and organizations that facilitate the implementation of HS. Negative experiences with new technology may undermine patients’ confidence, skill, and willingness to engage with systems such as HS [35].

Participants in our study reported high engagement in managing their own health and health care (as measured by PAM) with little variability, which limited our ability to study the relationship between patient activation and HS adherence. We found no relationship between age or gender and the use of HS, potentially suggesting that neither of these factors is a barrier to the implementation of HS. We did not directly measure participants’ experience with technology, and an additional investigation between comfort with technology use and HS adherence is warranted. However, the high level of acceptability of HS and patients’ willingness to overcome usability barriers (barring technical issues such as transmission failure) may reflect participants’ self-reported high levels of engagement. Patients with cancer have indicated a willingness to engage in remote monitoring when it potentially adds value to care [36,37].

Including end users in the technology development and modification process can enhance the potential for adoption and successful adherence to remote monitoring platforms. Participants in our study had suggestions for improving the experience of performing HS, including goal setting and feedback to ensure adherence of use. This type of feedback was not present in our study but easily implementable within the GoHome ecosystem in various forms, such as *CareTexts* directly to patients’ phones or tablets. Such a strategy may be useful to further reduce the burden of HS on the monitoring team [38], and others have found that implementing SMS text messaging into home spirometry programs may increase adherence [39]. We found that once participants were trained in spirometry, remote data monitoring was not time intensive, requiring approximately 20 minutes/patient every month [40].

Although our work provides evidence that trial participants viewed HS favorably, it remains to be seen whether this would also be true for patients in settings outside of tertiary care cancer centers. Notable differences include the possibility that HS may not be universally covered by payers, which may reduce enthusiasm for monitoring; that access to experts in pulmonary complications of AHCT may not be readily accessible; and that no data exist as to whether prompt interventions after pulmonary decline using real-world HS result in reductions in the incidence of BOS. In particular, the latter highlights the need for a randomized controlled trial comparing early interventions using HS to screen for impairment with the usual standard of care as it is possible that (1) early interventions do not reduce the rates of subsequent BOS and (2) that early interventions expose trial participants to unnecessary procedures, and thereby, unnecessary risks and costs. Therefore, despite positive patient perceptions toward HS, more definitive studies are necessary to prove that HS has a clinical role in detecting and treating BOS after AHCT.

Our study contributes to a growing body of research on remote patient monitoring in oncology. Notably, remote monitoring of electronic PROs, specifically cancer treatment–related symptoms, has been associated with improved quality of life and increased survival when providers intervene in response to worsening symptoms for the prevention of adverse downstream outcomes [41]. However, few studies in oncology have evaluated remote monitoring of biometric outcomes (eg, HS, temperature, blood pressure, weight, saturation of peripheral oxygen, and activity), with or without electronic PROs, using noninvasive digital technology [42]. In contrast, there has been greater progress in evaluating remote monitoring of biometric outcomes and PROs in respiratory, cardiovascular, and metabolic diseases and weight management [43]. Findings from studies in chronic conditions other than cancer are consistent with ours; namely, the data feedback loop wherein remotely captured patient data are enacted upon by providers is critical to affecting patient health outcomes [43]. Patient engagement with remote monitoring, as reflected in adherence to use, may be a critical limitation with regard to the accuracy and fidelity of the collected data [42]. Thus, addressing the technological barriers identified in this study that may affect adherence may optimize the value of remote monitoring.

### Strengths and Limitations

A major strength of this study was the use of a longitudinal mixed methods design using the same participants in repeated one-on-one qualitative interviews at multiple time points. This enabled us to understand how the feasibility and acceptability of HS use evolved over time. To the best of our knowledge, this is the first study to evaluate HS using this approach and the first to evaluate HS in patients with AHCT. Another strength is the use of a theoretical model to guide data analysis, thus allowing the sense-making process to be explicit. Finally, to our knowledge, this is the first study to investigate in depth the patient experience of participating in an HS program.

The results of this study should be taken in light of certain limitations. Our study population was predominantly White and English speaking; therefore, our results must be interpreted in the context of the limited cultural and racial diversity of the study cohort and may limit external validity to more diverse patient populations. In particular, although study devices were provided to all participants regardless of background, including providing tablets to participants without a smartphone, it is possible that further barriers to participation existed among participants of color. Participants generally had high PAM scores, which reduced our ability to associate patient activation with other measurements. The higher PAM scores may suggest that patients who were less likely to have higher levels of engagement in self-care declined study participation, although we cannot confirm this. However, we did not interview or measure PAM scores in patients who did not enroll in this study; therefore, we cannot comment on whether our cohort’s perspectives are reflective of all recipients of AHCT. We did not directly measure technological or health literacy and, therefore, cannot ascertain whether patients with low technological literacy require more instruction or supervision early in the monitoring period to ensure future adherence. As we did not comprehensively analyze open-ended patient responses in real time, we were unable to suggest modifications to the commercial spirometer used in this study. Finally, study attrition limited our ability to perform longitudinal assessments completely.

### Conclusions

In conclusion, we have established that patients found HS acceptable and easy to use. Simple technical and programmatic modifications were identified by the patients, which would improve the quality of HS implementation. Wider implementation of HS would benefit recipients of AHCT as well as other patients at risk for lung disease, particularly during crises such as the COVID-19 pandemic, where access to health care may be limited for various reasons. Given the limited data on the feasibility and acceptability of remote patient monitoring in oncology, these findings offer support for future research aimed at integrating remote monitoring technology to improve patients’ experiences and outcomes during acute cancer care. Future work is necessary to determine the efficacy of HS performed in the real world as a means of detecting and treating BOS and other pulmonary complications of AHCT.

## Abbreviations

AHCT: allogeneic hematopoietic cell transplantation
BOS: bronchiolitis obliterans syndrome
FEV_1_: forced expiratory volume in 1 second
GVHD: graft-versus-host disease
HS: home-based spirometry
PAM: patient activation measure
PRO: patient-reported outcome
REDCap: Research Electronic Data Capture
UTAUT: Unified Theory of Acceptance and Use of Technology
